# Hearing Loss &Tinnitus – Reducing the Risk of Cognitive Decline

**DOI:** 10.1002/alz70856_105797

**Published:** 2026-01-09

**Authors:** Keith N Darrow

**Affiliations:** ^1^ Worcester State University, Worcester, MA, USA

## Abstract

**Background:**

Hearing loss and tinnitus are potentially modifiable risk factors for cognitive decline and dementia^1,2,3^. Research indicates that untreated hearing loss contributes to social isolation^4^, increased cognitive load^5^, and neural atrophy^6^—key drivers of cognitive impairment^7,8^. Similarly, tinnitus, often linked to auditory neuropathy and dysfunction^9^, has been associated with an increased risk of neurologic disorders^10^, including Parkinson's and cognitive decline, particularly in adults over 60^11^. While hearing loss treatment has been suggested to slow cognitive decline^12,13^, the underlying mechanisms remain unclear and likely multifactorial. Emerging evidence suggests that early intervention for hearing loss and tinnitus may preserve cognitive resources^14,15^.

**Method:**

Cognitive function was assessed using F.D.A. cleared Cognivue Thrive technology. Subjects were fifty mid‐life subjects and recordings were made pre‐ and post‐treatment (day 0, 30 days, and 12 months). Measures included memory, executive function, visuospatial memory, processing speed, and reaction time. Participants received prescription hearing aid treatment, and cognitive assessments were conducted at each data collection point.

**Results:**

Significant improvements in memory, executive function, and visuospatial memory were observed at both the 30‐day and 12‐month follow‐up assessments. These findings suggest that auditory stimulation and intervention may enhance cognitive resilience by mitigating the effects of auditory deprivation on neural function.

**Conclusions:**

This study highlights the neurophysiological mechanisms driving cognitive improvements following hearing aid treatment, emphasizing the role of auditory stimulation in neural plasticity and cognitive resilience. By integrating clinical findings with real‐world outcomes, this research presents a compelling case for hearing healthcare as a critical strategy in global dementia prevention. Attendees will gain insight into the brain‐hearing connection, the cognitive benefits of prescription hearing aid treatment, and actionable strategies for reducing dementia risk through patient education and early intervention.

